# Medical Graduations

**Published:** 1835

**Authors:** 


### VI. Medical Graduations.

At the late public Commencements, the degree of M. D. was liberally conferred.

The number of graduates in Transylvania University was 82: in the University of Pennsylvania 130: in the Jefferson Medical College 58: in the University of Maryland 50.

The Medical College of Ohio, sent forth 27. Of whom one was lately a "steam doctor," and other a graduate of the University of Pennsylvania, who, not having faith in the permanence of our ancient Alma Mater, as she has lately had some slight convulsions, thought it prudent to obtain the diploma of a Faculty, which promises to enjoy great stability.

